# Hepatocyte Growth Factor Modulates Corneal Endothelial Wound Healing In Vitro

**DOI:** 10.3390/ijms25179382

**Published:** 2024-08-29

**Authors:** Merle Tratnig-Frankl, Nikolaus Luft, Guiseppe Magistro, Siegfried Priglinger, Andreas Ohlmann, Stefan Kassumeh

**Affiliations:** 1 Department of Ophthalmology, LMU University Hospital, Ludwig-Maximilians University Munich, Mathildenstrasse 8, 80336 Munich, Germany; merle.tratnigfrankl@meduniwien.ac.at (M.T.-F.); andreas.ohlmann@med.uni-muenchen.de (A.O.); 2 Department of Ophthalmology and Optometry, Medical University Vienna, AKH Vienna, Währinger Gürtel 18–20, 1090 Vienna, Austria; 3 Department of Urology, Asklepios Westklinikum Hamburg GmbH, Suurheid 20, 22559 Hamburg, Germany

**Keywords:** wound healing, endothelial cells, epithelial cells, cornea, functionality, skin, barrier

## Abstract

In this study, we assessed the impact of hepatocyte growth factor (HGF) on corneal endothelial cells (CECs), finding that HGF concentrations of 100–250 ng/mL significantly increased CEC proliferation by 30%, migration by 32% and improved survival under oxidative stress by 28% compared to untreated controls (*p* < 0.05). The primary objective was to identify non-fibrotic pharmacological strategies to enhance corneal endothelial regeneration, addressing a critical need in conditions like Fuchs’ endothelial dystrophy (FED), where donor tissue is scarce. To confirm the endothelial nature of the cultured CECs, Na^+^/K^+^-ATPase immunohistochemistry was performed. Proliferation rates were determined through BrdU incorporation assays, while cell migration was assessed via scratch assays. Cell viability was evaluated under normal and oxidative stress conditions using WST-1 assays. To ensure that HGF treatment did not trigger epithelial-mesenchymal transition, which could lead to undesirable fibrotic changes, α-SMA staining was conducted. These comprehensive methodologies provided robust data on the effects of HGF, confirming its potential as a therapeutic agent for corneal endothelial repair without inducing harmful EMT, as indicated by the absence of α-SMA expression. These findings suggest that HGF holds therapeutic promise for enhancing corneal endothelial repair, warranting further investigation in in vivo models to confirm its clinical applicability.

## 1. Introduction

The corneal endothelium, the innermost layer of the cornea comprising a single layer of cells, plays a crucial role in maintaining corneal transparency by facilitating hydration and active water transport [1,2], and its regenerative capacity postnatally is constrained [3]. Fuchs’ endothelial dystrophy (FED) is a prevalent corneal endothelium disease characterized by reversible corneal opacification caused by the accumulation of extracellular material on the Descemet’s membrane and the formation of guttae, accompanied by the gradual loss of corneal endothelial cells. CECs are responsible for actively pumping water out of the cornea, and their loss ultimately results in the development of corneal edema and a diffuse opacity of the cornea. Patients suffering from FED experience increasing photosensitivity and a decline in visual acuity [4]. 

The Descemet Membrane Endothelial Keratoplasty (DMEK) is currently the preferred surgical technique for treating FED due to its minimally invasive and sutureless nature. This corneal transplantation method necessitates the use of human donor material and was first introduced and established by Melles et al. in 2006 [5]. The procedure involves removing the remaining endothelium and Descemet membrane together, followed by the placement and fixation of a human corneal transplant onto the recipient’s cornea [5,6]. Once the transplant has been successfully attached, the donated endothelial cells migrate onto the recipient’s cornea, resulting in a clear cornea and a significant improvement in the patient’s visual acuity [7].

The global challenge posed by the supply of corneal transplants is currently one of the most pressing issues in the field of ophthalmology. According to statistics from 2012, nearly 200,000 corneal transplants were performed, of which 39% were necessary for the treatment of FECD [8]. The high cost and complexity associated with the provision of donor corneas means that more than half of the world’s population lacks access to this type of transplant [8]. Furthermore, the risk of a graft detachment, the most common complication, with a frequency of approximately 9.9% of all interventions, requires an additional surgical intervention [9]. To address these challenges and improve patient outcomes, there is a critical need for effective pharmacological treatments that can promote corneal regeneration and reduce dependence on donor tissues.

Various signaling pathways, including cytokine-mediated ones, are known to impact the activation of corneal endothelial cell proliferation and migration [10,11,12]. Thus, it is of high interest to identify substances that indirectly activate signaling pathways to promote corneal endothelial cell proliferation and migration, without causing fibrosis. One such substance group are Rho-Kinase inhibitors, which have been shown to promote cell proliferation and migration while having little or no effect on EMT acting through GTPases and activating the MLCK downstream signaling [13]. Identifying further substances, acting through different cell cycle pathways could provide a reliable therapy alternative for corneal endothelial damage.

Hepatocyte growth factor (HGF) is a cytokine that plays a crucial role in cell cycle regulation, tissue regeneration and wound healing [14,15,16]. It is produced by mesenchymal cells and acts on epithelial cells, particularly hepatocytes, to promote cell proliferation, survival and migration. It has been successfully shown that HGF acts through to the so-called c-Met receptor which is expressed on the surface of a variety of cells, including liver cells, kidney cells, immune cells and endothelial cells [17,18]. The binding of HGF to c-Met activates multiple intracellular signaling pathways, including the PI3K/Akt, MAPK/ERK, and STAT3 pathways, which regulate cell growth, survival and migration [19]. The expression of c-Met and HGF in corneal endothelial cells have been proven in several studies [20,21]. While the cell cycle activating effects of HGF have been researched extensively in other tissues, studies on the impact of HGF on the corneal endothelium are limited [20,22,23,24]. Topical details in dosage and effects have not been examined prior to our study. Kimoto et al. have investigated synergistic effects of HGF and L-Asc and confirmed an increase in CEC proliferation of these reagents combined [25]. Several reports further proved the proliferative capacity of HGF on the corneal epithelium and stroma [26,27,28] and similarly on endothelial cells of blood vessels as early as 1992 [22], which sparked our interest in remaining research gaps on the effects of HGF on the corneal endothelium.

Previous studies have further shown that HGF activates Smad7, which is known to counteract TGF-β, a potent inducer of EMT in other tissues [29,30]. Sumioka et al. describe the prevention of fibrosis in the corneal endothelium through overexpression of Smad7, fueling the interest to investigate similar antifibrotic effects through HGF [31].

Existing research on HGF and its properties has led us to explore the growth factor as a potential pharmacological agent in corneal wound healing, focusing on its proliferative effects on porcine corneal endothelial cells. We further investigate its impact on cell viability and migration, in addition to examining how HGF influences cell morphology and its role in modulating oxidative stress in vitro.

## 2. Results

### 2.1. Morphology and Immunohistochemistry of Corneal Endothelial Cells

**Figure 1:**
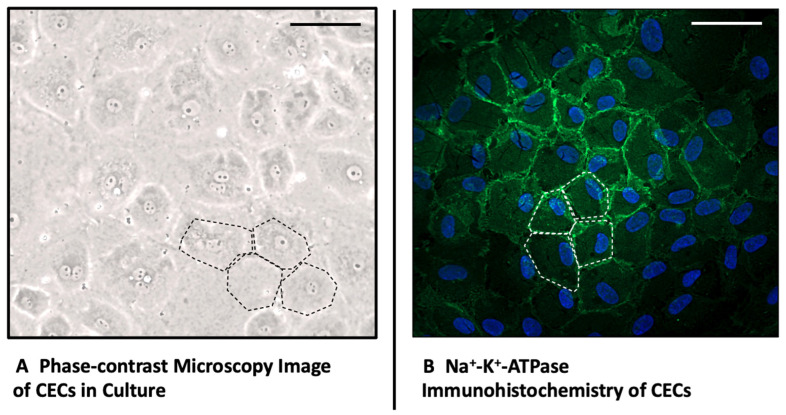
Morphology and Immunohistochemistry of Corneal Endothelial Cells. (**A**) Morphology of swine corneal endothelial cells in culture in Phase-contrast Microscopy, 20× magnification; (**B**) Immunohistochemistry of Na^+^-K^+^-ATPase and DAPI of cultured swine endothelial cells, 20× magnification. The dotted line exemplifies the pathognomonic cobble stone pattern of CECs in a tight endothelial monolayer, Scale Bars 40 μm.

To confirm the origin of the isolated corneal endothelial cells (CECs) from fresh swine bulbi, we performed immunostaining for Na+-K+-ATPase, a marker specific to the corneal endothelial cell membrane [32]. Figure 1 clearly shows that the monolayer of cells stained positively for Na+-K+-ATPase, displaying the characteristic hexagonal cell morphology that forms the typical cobblestone pattern associated with corneal endothelium [33]. This pattern, observed in both images, is indicative of healthy corneal endothelial cells and is maintained by tight junctions, as highlighted by the dotted lines in the images.

### 2.2. HGF Enhances Corneal Endothelial Cell Migration

**Figure 2:**
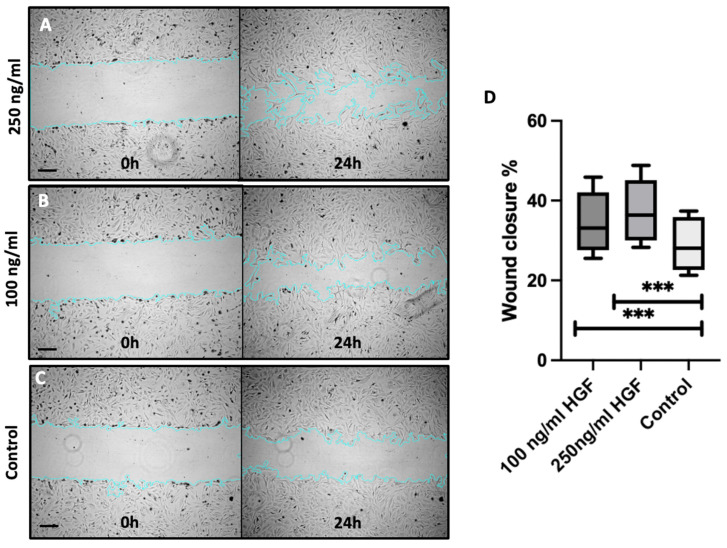
HGF Enhances Corneal Endothelial Cell Migration. (**A**–**C**) Representative images of the cell scratch assay under the treatment with different concentrations of HGF at start and after 24 h. Blue Line represents the Scratch border considered for measurements. (**D**) Analysis of the wound closure as a percentage of the migrated area. *n* = 154 total scratches considered for the analysis, 7 independent experiments. *** *p* < 0.0001 for both treatment groups compared to the control, Scale Bars 100 μm, light grey: untreated control, mid-grey: 250 ng/mL HGF, dark grey: 100 ng/mL HGF.

Figure 2 illustrates the progression of wound closure over 24 h for each experimental group, with images captured at 0 h (left) and 24 h (right). Panel A represents the group treated with 250 ng/mL HGF, panel B corresponds to the group treated with 100 ng/mL HGF and panel C depicts the control group without HGF treatment. After 24 h, approximately 31% of the scratch area was covered by migrating corneal endothelial cells (CECs) in the control group. In contrast, the 250 ng/mL HGF-treated group exhibited 41% wound closure, while the 100 ng/mL HGF group demonstrated 37% closure. Quantitative analysis confirmed a statistically significant enhancement in wound closure with HGF treatment, showing a 32% increase (*p* < 0.0001) at 250 ng/mL and a 20% increase (*p* < 0.0001) at 100 ng/mL, as depicted in graph D.

### 2.3. HGF Increases Corneal Endothelial Cell Viability and Proliferation

**Figure 3:**
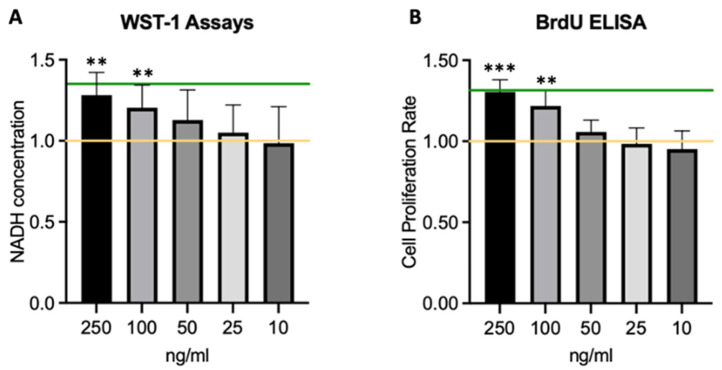
HGF Increases Corneal Endothelial Cell Viability and Proliferation. (**A**) WST-1 viability and (**B**) BrdU-ELISA proliferation assay results, measured after a 72 h-incubation period with HGF with 250 ng/mL, 100 ng/mL, 50 ng/mL, 25 ng/mL, 10 ng/mL, respectively. Target lines indicate the average control values; yellow = negative control, green = 10% FCS control, ** *p* < 0.01; *** *p* < 0.001.

Figure 3 demonstrates that incubation with exogenous HGF increases both cell proliferation in BrdU-ELISA assays and viability of CECs in WST-1 viability assays in a dose-dependent manner. Specifically, HGF treatment at 250 ng/mL increased cell viability by 28% compared to untreated control cells in WST-1 assays (Figure 3A) and up to 30% proliferation rates in BrdU ELISA assays (Figure 3B) when treated with 250 ng/mL for 72 h. These results were statistically significant (*p* < 0.05) compared to the control. Results from 13 independent WST-1 assays and 10 BrdU-ELISA assays were analyzed.

### 2.4. HGF Protects Corneal Endothelial Cells from Oxidative Stress

**Figure 4:**
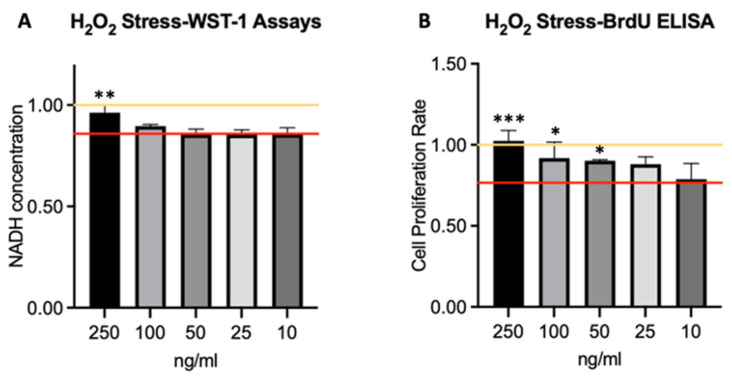
HGF Protects Corneal Endothelial Cells from Oxidative stress. (**A**) H_2_O_2_ WST-1 viability and (**B**) H_2_O_2_ BrdU-ELISA proliferation assay results, measured after a 72 h-incubation period with HGF with 250 ng/mL, 100 ng/mL, 50 ng/mL, 25 ng/mL and 10 ng/mL, respectively. Target lines indicate the average control values; yellow = negative control, red = H_2_O_2_ control, * *p* < 0.1; ** *p* < 0.01; *** *p* < 0.001.

Results of the potential effects of HGF on oxidative stress on swine CECs are shown in Figure 4. We exposed cells to hydrogen peroxide (H_2_O_2_) stress, depicted with the red control line, and measured the impact of HGF treatment on cell viability using the WST-1 assay and proliferation using BrdU ELISA. Our results show that treatment with H_2_O_2_ significantly decreased cell viability by about 14% in seven independent WST-1 assays in (Figure 4A) and 23% in the BrdU proliferation rate (Figure 4B), compared to untreated control cells (*p* < 0.05) in six different assays as shown in the diagrams. However, additional treatment with HGF at a higher concentration of 250 ng/mL for 72 h significantly neutralizes the negative influence of H_2_O_2_-stressed cell viability compared to untreated stressed cells (*p* < 0.05) reaching 96% of the control levels in the WST-1 and even 102% in the BrdU ELISA. This suggests that exogenous HGF increases viability and proliferation in the presence of oxidative stress and (partially) eliminates the effect imposed by the addition of H_2_O_2_.

### 2.5. HGF Does Not Induce α-SMA Expression in Corneal Endothelial Cells

**Figure 5:**
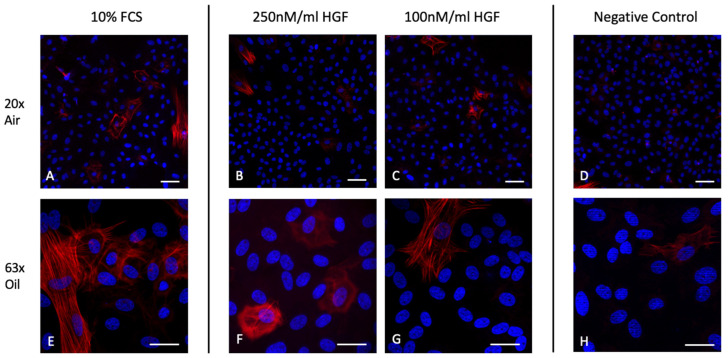
HGF Does not Induce α-SMA expression in Corneal Endothelial Cells. All images show the immunohistochemistry of corneal endothelial cells stained for DAPI and α-SMA after incubation with different concentrations of HGF or 10% FCS. Images (**A**–**D**) show a magnification of 20×, allowing a greater overview of the cell culture. The images below (**E**–**H**) show a larger magnification and detail of 63× in oil immersion, Scale Bars Images (**A**–**D**) 40 μm, (**E**–**H**) 20 μm.

Figure 5 shows the immunohistochemistry of corneal endothelial cells stained for DAPI and α-SMA, which is a component of the intracellular cytoskeleton and is upregulated in the process of fibrogenesis and correlates with the activation of myofibroblasts, thus serving as a marker for EMT [34]. Images (Figure 5A) and (Figure 5E) show an increased staining for the intracellular skeletal marker, compared to the negative control images (Figure 5D) and (Figure 5H). CECs treated with HGF show isolated positive stains for α-SMA, however comparably less than the control treated with 10% FCS. CECs in images (Figure 5B) and (Figure 5F), which were incubated with 250 ng/mL for 72 h show no significant difference in α-SMA expression to lower concentrations of 100 ng/mL HGF in images (Figure 5C) and (Figure 5G).

## 3. Discussion

Efforts to regenerate the corneal endothelium encompass a wide range of research endeavors worldwide, including the exploration of pluripotent stem cells, the utilization of biomaterials or the pharmacological induction of cell proliferation and migration [35,36,37,38,39].

Based on our findings, we assert that HGF exhibits a specific capacity to stimulate in vitro corneal endothelial wound healing, primarily through various cell cycle pathways. This conclusion is supported by (1) the demonstrated increase in the proliferation and cell viability with higher concentrations of HGF, (2) its ability to mitigate the adverse effects of H_2_O_2_ supplementation, (3) the observed acceleration of wound healing attributed to enhanced cell migration and (4) the absence of any significant detrimental impact on endothelial cell morphology. However, our study has several limitations that should be acknowledged. One significant limitation is the reliance on in vitro models, which may not fully replicate the complexity of the in vivo corneal environment. Further studies in more physiologically relevant systems, including animal models, are necessary to validate these findings.

Damages in the corneal endothelium through diseases such as FECD disrupt the normal function of CECs, leading to their gradual loss. Due to limited proliferative capacity, compromised fluid regulation and the development of corneal edema, ultimately impacting visual acuity are the result [40]. The pursuit of enhancing corneal endothelial proliferation has prompted investigations into various molecular targets, e.g., to inhibit Transforming Growth Factor-Beta (TGF-β) ROCK inhibitors, mitogen-activated protein kinase (MAPK) inhibitors etc. [41,42,43]. However, current substances under investigation show distinctive limitations. TGF-β has been shown to stimulate fibrosis and transdifferentiation of cells, thus triggering EMT and increasing inflammation [44,45,46]. ROCK inhibitors seemingly show dose-related adverse effects in higher dosage and influence the aqueous humor outflow, potentially affecting ocular pressure, a concern that requires careful monitoring during treatment [18,47]. Our study clearly demonstrated that the addition of HGF in higher doses (e.g., 100 and 250 ng/mL) to CECs in culture had a stimulatory effect on cell cycle activities, indicated through an increased rate of proliferation. A potential explanation for the proliferative effects of HGF could be the activation of the following pathways. Current research states that endogenous HGF mediating their wound healing effects, at least in part via key pathways involved, include the Ras/Raf/MEK/ERK pathway, promoting cell proliferation; the PI3K/Akt pathway, supporting cell survival and growth; the JAK/STAT pathway, influencing gene transcription and cellular responses; the Wnt/β-Catenin pathway, contributing to tissue repair; and the NF-κB pathway, regulating inflammatory responses [48,49,50,51,52]. The convergence of these pathways underscores HGF’s multifaceted effects in promoting corneal endothelial cell regeneration. However, the precise molecular mechanisms of HGF still have to be fully understood and require further investigation. The variety of pathways potentially affected by HGF could also point out a lack of specificity to treat endothelial diseases.

Oxidative stress, arising from an imbalance between reactive oxygen species (ROS) production and antioxidant defenses, is a central factor in wound healing dynamics, impacting cellular signaling pathways during tissue repair, including NF-κB signaling for inflammation, VEGF signaling for angiogenesis and TGF-β signaling for extracellular matrix remodeling [53,54]. The positive impact observed in our experiments on viability and proliferation rates in CEC cultures supplemented with H_2_O_2_ upon the addition of HGF prompts an inquiry into the antioxidative capacity of HGF, potentially safeguarding CECs from oxidative stress. Multiple reports substantiate this notion [25,55,56]. However, further experiments, e.g., DPPH or Glutathione assays, gene expression analysis or the impact on mitochondrial redox function are necessary to undermine this thesis [57]. On the contrary, current research also suggests that HGF may modulate cellular responses to ROS rather than acting as a direct scavenger [56,58]. Its effects on cellular redox balance may involve intricate signaling pathways, such as increased cell survival rather than ROS neutralization, which cannot be distinguished by the chosen viability assays and potentially conflate results. While HGF’s involvement in regenerative processes may indirectly influence cellular responses to oxidative stress, attributing direct antioxidative effects to HGF may oversimplify its complex biological activities. This study is further limited as H_2_O_2_ cannot mimic the multifactorial genesis of oxidative stress in wound healing.

Since the corneal endothelium, once damaged, does not actively divide, the remaining healthy endothelial cells aim to compensate by migrating and enlarging to cover damaged areas, contributing to tissue repair. However, in cases of extensive damage to the cornea, such as in Fuchs Endothelial Dystrophy, with significant loss of endothelial cells, physiological migration alone proves insufficient for substantial functional recovery, necessitating additional interventions to restore the inner monolayer of the cornea. Current research attempts to target different stimuli to initiate migration in CECs. Rho-Kinase inhibitors such as Y-27632 influence the actin cytoskeleton dynamics, thus promoting migration [42,59,60]. Growth factors such as epidermal growth factor (EGF), platelet-derived growth factor (PDGF) or our examined growth factor HGF have all been shown to target signaling pathways involved in tissue repair such as Ras-Raf-MEK-ERK or more importantly for migration—the PI3K-Akt pathway [61]. HGF further enhances the expression of various extracellular matrix proteins, such as collagen and fibronectin [62], which are crucial for CEC adhesion and migration. An increase in extracellular matrix and scarring can, on the other hand, also trigger the epithelial mesenchymal transition of CECs and could thus impose a limitation on the use of HGF in CEC therapy. Therefore, it is important to develop treatments that can promote endothelial regeneration without triggering EMT.

Our results have successfully proven that the supplementation of HGF in concentrations of 100 to 250 ng/mL accelerates wound healing through migration. Further studies might elaborate the influence of HGF on CECs in a more physiological environment containing ECM molecules to mimic a natural microenvironment of the cornea, including cellular interactions with the underlying basement membrane, which plays a crucial role in endothelial cell migration and adhesion. Scratch migration assays are also limited in their informative value, as the migration results are confounded with cell proliferation on the wound edges, which makes it hard to isolate and measure the specific migratory behavior of cells without the interference of cell division.

As previously described, migration in a damaged corneal endothelium is accompanied by epithelial-mesenchymal transition (EMT), which involves the loss of typical cobblestone-like morphology and cell-cell adhesion [63]. Dysregulated EMT could impair the cells’ ability to maintain corneal transparency and regulate fluid balance effectively and result in corneal fibrosis and potentially lead to the formation of dense membranes inside the cornea and a subsequent loss of vision [64,65]. Lower HGF dosages have previously shown potential to suppress EMT by promoting the maintenance of the original phenotype and inhibiting the acquisition of mesenchymal characteristics [66]. This is often associated with the modulation of key signaling pathways, including those involving TGF-β and Wnt, which play crucial roles in the regulation of EMT [18]. However, the specific mechanisms and outcomes can vary depending on the context, concentration of HGF and the microenvironment of corneal endothelial cells. In line with these observations, following the incubation of CECs with HGF, we observed that the morphology of the CECs stained with the EMT marker α-SMA remained similar. α-SMA is known to indicate the acquisition of a myofibroblast-like state with increased contractility [34]. Petroll et al. have shown that CECs lose the tight junction protein ZO-1 and instead express α-SMA when injured [67]. Thus, α-SMA serves as a crucial marker for the mesenchymal transition and is associated with heightened production of ECM components, particularly collagens. Other extracellular markers such as collagens and fibronectin should be examined to assess cell adhesion and morphology [68]. However, our findings on the impact of HGF on EMT are limited, and no epithelial markers have been stained so far. Since basement membrane integrity also plays a crucial role in EMT and cell morphology, the impact of HGF also in a prolonged exposure on laminin and cadherin expression should further be investigated in vitro before advancing our studies to in vivo experiments. Quantifying the expression levels of mesenchymal and epithelial markers offers a more objective approach to evaluating the impact on EMT. Using an EMT inducer, (e.g., TGF-β) as a positive control could provide better context for interpreting the results.

## 4. Materials and Methods

### 4.1. Swine Corneal Endothelial Cells

#### 4.1.1. Animal Tissue and Cell Isolation

Swine eyes were obtained by MRT Leidmann GmbH, a local butchery in Munich, Germany. The swine globes were processed within 12 h after death and stored at 4 °C until further processing. Prior to storage or usage, they were washed in 7.5% Povidon-Iodide (B. Braun Melsungen AG, Melsungen, Germany) for five minutes and then rinsed three times with Dulbecco’s Phosphate Buffered Saline (PBS; Sigma Life Science, St. Louis, MO, USA) for five minutes. A corneoscleral disc was excised using a scalpel and scissors. The corneoscleral disc was gently rinsed in PBS and then placed on a plate with the epithelium facing down, forming a cup. This cup was then filled with 100–150 μL 2.5% trypsine in PBS (Biochrom GmbH, Berlin, Germany) and incubated at 37 °C for seven minutes. The trypsine was neutralized using 300–400 μL DMEM (Dulbecco’s MEM; Biochrom GmbH, Berlin, Germany), supplemented with 10% fetal calf serum (Biochrom GmbH, Berlin, Germany), 50 IU penicillin/mL and 50 µg streptomycin/mL (Penicillin-Streptomycin; Sigma Life Science, St. Louis, MO, USA). The remaining medium in the cup was then pipetted repeatedly up and down in order to gently detach the corneal endothelial cells and transferred to a vial. The detachment of the endothelial cells was observed under a stereo microscope (Stemi 508; Carl Zeiss AG, Jena, Germany). The medium containing the cells was then centrifuged at 800 rounds per minute for five minutes at room temperature, and the cell pellet was transferred to a well with medium.

#### 4.1.2. Cell Culture

To increase the attachment of isolated endothelial cells in culture, an attachment factor (Cell Systems, Kirkland, WA, USA) was pipetted onto well plates/cell culture flasks and incubated for one minute. The remaining fluid was removed. The attachment factor dries instantly and is ready for immediate use. The cells are cultured in DMEM, supplemented with 10% fetal calf serum and 2% penicillin/streptomycin per ml. The cell culture flasks and well plates are stored in an incubator at 37 °C and 5% carbon dioxide. The freshly isolated cells were placed in a 6-well plate and took variable time to grow until confluence, a week on average. The cells were then passaged and cultured in a T-25 flask with a seeding density of 1.5 to 2.0 × 10^5^ cells. Passages (P) used for experiments were between P1 and P4 maximum, due to increased epithelial-mesenchymal-transition in further passages.

### 4.2. Immunohistochemistry

#### 4.2.1. Na^+^-K^+^-ATPase Staining

To verify the origin of the cells from the corneal endothelium, Na^+^-K^+^-ATPase staining was used to mark the cell membrane. The Na^+^-K^+^-ATPase chosen for immunostaining is specific to the corneal endothelium [32]. This step was crucial to verify that there was no contamination with other corneal cells, such as epithelial cells, which have much higher regenerative capacity. Cells used for staining originated from passage zero. The cells were cultured on glass tiles until 70% confluency and subsequently fixed in 4% paraformaldehyde (PFA, Carl Roth GmbH & Co. KG, Karlsruhe, Germany) for five to seven minutes, followed by three PBS rinses for five minutes. Cells were then blocked in PBS + 3% bovine serum albumin (BSA, Thermo Fisher Scientific, Waltham, MA, USA) + 0.1% Triton X-100 (Merck KGaA, Darmstadt, Germany) for one hour at room temperature. The blocking solution was again followed by three washing cycles for five minutes. The cells were incubated in an alpha-1 subunit rabbit Na^+^-K^+^-ATPase antibody conjugated with mouse Alexa Fluor 488 antibody (Clone C464.6; Lot Number: 3218764, Merck KGaA, Darmstadt, Germany) overnight in a moist chamber at 4 °C. 1 μL antibody was diluted in 250 μL Blocking, which was diluted 1:10 in PBS. After a single rinse in PBS for five minutes, the cells were stained with 4′,6-Diamidino-2-phenylindol (DAPI, Thermo Fisher Scientific, Waltham, MA, USA) diluted 1:1000 in PBS for 10 min and washed once more for 5 min. The glass tiles with the stained cells were placed on objective slides using 5 μL mountant (ProLong Glass Antifade Mountant, Fisher Scientific GmbH, Schwerte, Germany) and stored in a moist chamber at 4 °C until analysis was performed using an Axio Observer 3 (Zeiss, Jena, Germany) and the ZEN software (Zeiss, Jena, Germany).

#### 4.2.2. Epithelial-Mesenchymal-Transition Staining

To observe the extent of epithelial-mesenchymal-transition, alpha smooth muscle antigen/ACTA 2 (α-SMA) was stained to evaluate the morphology of the cells, as it serves as a marker of myofibroblast formation [69]. Physiologically, endothelial cells have a hexagonal shape and present themselves in a so-called cobble stone pattern [33]. When transitioning into fibroblasts, the cell shape is elongated and thinned, which impairs the corneal endothelial function. Thus, corneal endothelial cells in different HGF concentrations (human recombinant HGF from *E.coli*; Cat. Number: H9268, Merck KGaA, Darmstadt, Germany) were stained with α-SMA mouse monoclonal antibody (Clone 1A4; Catalog Number: M0851, Dako/Agilent Technologies, Hamburg, Germany) and a secondary IgG antibody goat anti-mouse Alexa Fluor 555 (Catalog Number: A21422, Thermo Fisher Scientific, Waltham, MA, USA) to assess the degree of scarring. The fixation and blocking protocol were conducted as previously described.

### 4.3. Scratch Migration Assay

Scratch migration assays were carried out according to a previously published protocol [70]. Cells were densely cultured until 90 to 100% confluency on a 6-well-plate. The cells were then deprived of FCS supplemented culture medium and placed in FCS-free DMEM for six hours. Five vertical scratches were performed in each of the six wells using a 100 μL pipette tip. The culture medium was subsequently removed and replaced with DMEM, containing different HGF treatment concentrations in DMSO or just DMSO serving as the control. Thus, two wells contained a control, two wells were incubated with a 100 ng/mL HGF concentration and another two with 250 ng/mL. The process of cell migration was recorded at 0, 12, 24, 48 and 72 h. The size of the wound area was documented again using the previously mentioned Zeiss Axio Observer 3 and ZEN software before and 24 h after treatment with HGF. The wound area was measured using Image J Version 1.52k (NIH, Bethesda, MD, USA) and calculated as relative wound closure.

### 4.4. Cell Viability and Cell Proliferation

To assess cell viability, a colorimetric dye reduction assay was performed according to the manufacturer’s protocol (WST-1; Roche Diagnostics GmbH, Mannheim, Germany). Swine CECs were seeded with a density of 1.4 × 10^4^ cells/cm^2^, equaling 5000 cells per well onto a 96-well plate and incubated for 12 h before being deprived of FCS for another 12 h prior to treatment with different HGF concentrations for 72 h. Subsequently, 67 μL WST-1 dilution (100 μL WST-1 per mL DMEM) was then added to each well the cells and incubated for 60 min before absorbance was measured at 450 nm with a reference at 690 nm using a SpectraMax 190 ELISA reader (Molecular Devices, Sunnyvale, CA, USA).

The proliferation rate of the swine CECs was determined using a 5-bromo-2′-deoxyuridine (BrdU) ELISA as described by the manufacturer (Roche Diagnostics GmbH, Mannheim, Germany). The cells were seeded with a density of 1.4 × 10^4^ cells/cm^2^, equaling 5000 cells per well onto a 96-well plate and incubated overnight to allow complete adherence before treatment. After 12 h, cells were deprived of FCS for 12h and then labelled with BrdU labeling solution together with the treatment medium (1 μL labeling reagent in 1 mL DMEM) incubating for 72 h. To assess the effects of HGF on cell proliferation, the cells were fixed after the 72 h time period and incubated with anti-BrdU antibodies in accordance with the manufacturer’s instructions. The proliferation rate was quantified by measuring the absorbance at 450 nm with a reference at 690 nm using a spectrophotometer (Spectramax 190, Molecular Devices, Sunnyvale, CA, USA) after 60 min.

### 4.5. Oxidative Stress Model

The WST and BrdU-ELISA were modified to assess oxidative stress. All HGF-treated wells additionally contained 1:1000 H_2_O_2_ in DMEM, resulting in oxidative stress for the corneal endothelial cells, simulating the oxidative stress existent in a wounded tissue [71]. One control group contained DMEM only, whereas the second control contained H_2_O_2_ and DMEM. The first control without H_2_O_2_ serves as the standard, untreated cell proliferation rate. The second control shows the level of stress caused by the H_2_O_2_, allowing to assess the resulting decrease in the proliferation rate through H_2_O_2_. The subsequent HGF conditions were added to investigate its effect on CECs presented with oxidative stress.

### 4.6. Statistical Analysis

All results are expressed as mean ± SEM. A one-way analysis of variance (ANOVA) was performed to compare the mean variables of more than two groups. A Tukey post-hoc test followed for data that meet or do not meet the criteria of the assumption of homogeneity of variances, respectively. To depict significant changes over time (wound healing assays), a two-way ANOVA with Geisser-Greenhouse correction was conducted. A *p*-values less than 0.05 was considered statistically significant. Data analysis was conducted using the GraphPad PRISM 10 Software (GraphPad Software Inc., San Diego, CA, USA)

## 5. Conclusions

Loss of the corneal endothelium is a severe vision-threatening complication of several diseases such as FECD, bullous keratopathy, neuropathic ulcers or keratitis [72]. This study demonstrates that hepatocyte growth factor (HGF) significantly enhances the proliferation, migration and viability of porcine corneal endothelial cells (CECs) in vitro, while also providing protection against oxidative stress. Importantly, HGF did not induce epithelial-mesenchymal transition (EMT) as indicated by the absence of α-SMA expression, suggesting that it promotes corneal endothelial wound healing without triggering fibrosis. These findings highlight the therapeutic potential of HGF as a pharmacological agent for corneal endothelial regeneration, offering a promising alternative to traditional treatments that rely on donor tissues. Future in vivo studies are warranted to further explore the clinical applicability of HGF in treating corneal endothelial dysfunction, particularly in conditions like Fuchs’ endothelial dystrophy (FED), where donor tissue availability remains a critical challenge.

## Data Availability

The original contributions presented in the study are included in the article. Further inquiries can be directed to the corresponding author.
